# Novel Group of Imidazole Derivatives as Atypical Selective Cyclooxygenase-2 Inhibitors: Design, Synthesis and Biological Evaluation

**Published:** 2018

**Authors:** Azin Kiani, Elham Rezaee, Sayyed Abbas Tabatabai

**Affiliations:** *Department of Pharmaceutical Chemistry, School of Pharmacy, Shahid Beheshti University of Medical Sciences, Tehran, Iran.*

**Keywords:** COX-2 inhibitor, Imidazole derivatives, Atypical, Synthesis, Docking

## Abstract

In this study, a new series of 5-substituted 1-benzyl-2-(methylsulfonyl)-1-H-imidazole with atypical structure-activity relationship was designed, synthesized, and biological evaluated as selective cyclooxygenase-2 inhibitors. Docking studies revealed that although the pharmacophoric substitute of the compound 5b, methylsulfonyl group, has been directly attached to the central ring, it is in the same direction of the sulfonamide group of Celecoxib, a known selective cyclooxygenase-2 inhibitor. Therefore effective hydrogen binding with Arg513 is established. Also, additional hydrogen binding could form between NH of anilino moiety of the 5b and Arg120. All of the compounds had selective inhibitory activity against cyclooxygenase-2 in micromolar concentrations comparable with the reference, Celecoxibe. Finally, compound 5b with the selectivity index 115 and IC_50_ of 0.71 µM against cyclooxygenase-2 was the most potent one.

## Introduction

**Figure 1:**
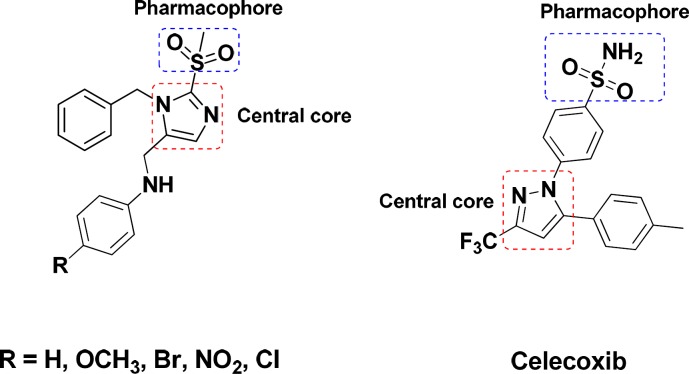
Chemical structures of the designed compounds compared with Celecoxibe, a known COX-2 inhibitor

Cyclooxygenase (COX) is an endogenous enzyme that plays a central role in biosynthesis of the important biological mediators, prostaglandins, from Arachidonic acid (1). The two most known isoforms of COX (COX-1 and COX-2) share about 60% amino acid sequence but are encoded by different genes and have different biological roles (2, 3). The constitutive form, COX-1, is expressed in normal physiologic condition to maintain homeostasis, gastric, renal blood flow, and platelet aggregation while the inducible form, COX-2, is expressed in pain and inflammatory conditions (4-6). Classic nonsteroidal anti-inflammatory drugs (NSAIDs) block both COX-1 and COX-2 non selectively and more tightly to COX-1 (7, 8) leading to the lack of the prostaglandins with normal physiological roles specially in long term use and consequently have several certain renal (9), gastrointestinal (10) and cardiovascular side effects (4, 11 and 12). These side effects prompted the development of selective COX-2 inhibitors with comparable efficacy and improved gastrointestinal safety (13, 14). The involvement of COX-2 in cancer development and neurodegenerative disease was previously evidenced. Therefore, selective COX-2 inhibitors are promising in the treatment of malignant and neurodegenerative disorders, such as adenocarcinoma, Alzheimer’s, and Parkinson’s disease (15-19). However, the cardiovascular risks such as myocardial infraction and thrombosis related to selective inhibition of COX-2 due to the depression of the biosynthesis of atheroprotective prostaglandin (PGI_2_) and not the pro-aggregatory and vasoconstrictor mediator thromboxane A_2_ derived from COX-1 (20) leaded to a withdrawal of Rofecoxib and Valdecoxib from the market (21, 22). Thus, the challenge persists to explore and evaluate selective COX-2 inhibitors with a mild tendency to COX-1 in order to reduce the cardiovascular side effects and enhance the safety profile along with addressing the unmet medical needs (22, 23). The majority of selective COX-2 inhibitors are diarylheterocycles with vicinal substitution attached to a mainly mono or bicyclic central ring (24-26). According to extent structure activity relationship (SAR) studies, the optimum COX-2 selectivity will be provided with a SO_2_NH_2_ or a SO_2_Me substituent at the para position of one of the phenyl rings (27-29). In continuance of our previous studies on five member heterocycle rings (30-37), in this study, a new structure-activity relationship is presented with an imidazole cycle as the central heterocyclic ring and unlike classic COX-2 inhibitors the pharmacophore of methylsulfonyl is attached to the central core (Figure 1). 

The docking study and biological evaluation were performed to clear the orientation of the synthesized compounds in the COX-2 active site and inhibitory activity of all compounds respectively.

## Experimental

*Molecular Modeling Studies*

Docking simulation was performed to predict interaction of compounds (5a–f) with COX-2 binding site. The high resolution crystal structure of COX with Celecoxib as a cognate ligand was retrieved from RCSB Protein Data Bank (PDB code: 6COX). The structures of the compounds were investigated using the Lamarckian genetic algorithm search method implemented in AutoDock 4.0 software. The receptor was kept rigid and ligands were allowed to be flexible. Polar hydrogens and Kollman united atom partial charges were added to the individual protein atoms. The HyperChem 8 software was used for energy minimization of each structure under MM+ method and AutoDockTools 4.0 version 1.5.6rc3 for conversion of file formats to pdbqt. A docking grid box was built with 40, 40, and 40 points in 24.4370, 22.8660, and 48.5210 directions and the number of generations and maximum number of energy evaluations was set to 100 and 2,700,000, respectively. The docking results were clustered with a root mean square deviation (RMSD) of 0.5 Å and evaluated by Pymol software (38-40).

*Chemistry*

All the chemicals and solvents were purchased from Merck or Aldrich Company and were used without further purification. Thin layer chromatography (TLC) was performed on commercially available Merck precoated plates (silica gel 60 F254, 0.25 mm). Melting points were obtained using the Electrothermal 9100 apparatus and are uncorrected. Infrared spectra were acquired on a Perkin Elmer 843 spectrometer. A Bruker FT-400 MHz instrument (Bruker Biosciences, USA) was applied to obtain ^1^HNMR spectra; DMSO-d_6_ was used as solvents. LC Mass spectra and elemental analysis were achieved by HPLC Agilent system and Costech (Italy) elemental analyzer respectively.

*(1-benzyl-2-mercapto-1H-imidazol-5-yl)methanol (* ***1*** *)*

Potassium thiocyanate (5.44 g, 55.79 mmol) , dihydroxyacetone (3.67 g, 40.74 mmol) and benzylamine (4 mL, 37.33 mmol) were refluxed at 55 °C for 18 h in 136 mL of water and glacial acetic acid (7/93). The mixture was alkalinized with NaOH 10% and extracted with chloroform. The resulting precipitates were formed after acidifying of the aqueous phase with hydrochloric acid and recrystallized from ethanol 96% to give 6.66 g (54.1%) of **1**. mp: 127 °C; IR: (KBr) ν (cm^-1^) 2739-3200 (O-H). ^1^HNMR (DMSO, 400 MHz) δppm: 4.14 (s, 2H, CH_2_OH), 5.29 (s, 1H, OH), 5.32 (s, 2H, benzyl), 6.87 (s, 1H, imidazole), 7.22 (d, *J* = 7.2 Hz, 2H, H_2_, H_6_-benzyl), 7.26 (t, *J* = 7.2 Hz, 1H, H_4_-benzyl), 7.32 (t, *J* = 7.2 Hz, 2H, H_3_, H_5-_benzyl), 12.04 (s, 1H, SH). LC-MS [M+1]^+^: m/z 221, [M+23]^+^: m/z 243, [M+39]^+^: m/z 259. Anal. Calcd for C_11_H_12_N_2_OS: C, 59.97; H, 5.49; N, 12.72. Found: C, 59.85; H, 5.51; N, 12.75. 

*(1-benzyl-2-(methylthio)-1H-imidazol-5-yl)methanol* ***(2)***

A solution of 1.1 g (5 mmol) intermediate **1** and 1.1 mL (5 mmol) sodium iodide in presence of 10 mL of NaOH 10% in ethanol was stirred at room temperature for one hour. The solvent was evaporated and the remnant was acidified and extracted with chloroform. The Aqueous phase was alkalinized with NaOH 10% to participate 0.81 g (69%) of **2**. mp: 106 °C; IR: (KBr) ν (cm^-1^) 2850-3200 (O-H). ^1^HNMR (DMSO,400 MHz) δppm: 2.44 (s, 3H, SCH_3_), 4.33 (s, 2H, CH_2_OH), 5.21 (s, 3H, OH, 2H benzyl), 6.93 (s, 1H, imidazole), 7.07 (d, *J* = 7.2 Hz, 2H, H_2_, H_6-_benzyl), 7.26 (t, *J* = 7.2 Hz, 1H, H_4_-benzyl), 7.34 (t, *J* = 7.2 Hz, 2H, H_3_, H_5_-benzyl). LC-MS [M+1]^+^: m/z 235, [M+23]^+^: m/z 257. Anal. Calcd for C_12_H_14_N_2_OS: C, 61.51; H, 6.02; N, 11.96. Found: C, 61.75; H, 5.96; N, 11.93.

*(1-benzyl-2-(methylsulfonyl)-1H-imidazol-5-yl)methanol* ***(3)***

Compound **2 (**1 g, 4.3 mmol) in THF was added to a solution of Oxon (6.22 g, 51.3 mmol) in water. The mixture was stirred at room temperature overnight and after evaporating THF, 0.62 g (54.1%) of compound **3** was participated. mp: 127 °C; IR: (KBr) ν (cm^-1^) 2848-3538 (O-H), 1144, 1327 (SO_2_). ^1^HNMR (DMSO,400 MHz) δppm: 3.28 (s, 3H, SO_2_CH_3_), 4.35 (s, 2H, CH_2_OH), 5.47 (s, 1H, OH), 5.62 (s, 2H, benzyl), 7.10 (d, *J* = 7.2 Hz, 2H, H_2_, H_6_-benzyl), 7.30 (m, 2H, imidazole, H_4-_benzyl), 7.36 (t, *J* = 7.2 Hz, 2H, H_3_, H_5_-benzyl). LC-MS [M+1]^+^: m/z 267, [M+23]^+^: m/z 289. Anal. Calcd for C_12_H_14_N_2_O_3_S: C, 54.12; H, 5.30; N, 10.52. Found: C, 53.96; H, 5.34; N, 10.60.

*1-benzyl-5-(chloromethyl)-2-(methylsulfonyl)-1H-imidazole* ***(4)***

Compound **3** (400 mg, 1.5 mmol) was refluxed in 2 mL (16.8 mmol) of thionyl chloride for 4 h. After evaporating thionyl chloride, 0.21 g of compound **4** (57%) was obtained. mp: 127 °C; IR: (KBr) ν (cm^-1^) 1154, 1347 (SO_2_). ^1^HNMR (DMSO,400 MHz) δppm: 3.33 (s, 3H, SO_2_CH_3_), 4.78 (s, 2H, CH_2_OH), 5.66 (s, 2H, benzyl), 7.10 (d, *J* = 7.2 Hz, 2H, H_2_, H6-benzyl), 7.31 (t,* J* = 7.2 Hz, 1H, H_4_-benzyl,), 7.34 (t, *J* = 7.2 Hz, 2H, H_3_, H_5_-benzyl), 7.40 (s, 1H, imidazole). LC-MS [M+1]^+^: m/z 285, [M+23]^+^: m/z 307. Anal. Calcd for C_12_H_13_ClN_2_O_2_S: C, 50.61; H, 4.60; N, 9.84. Found: C, 50.74; H, 4.56; N, 9.79.

*General procedure for the synthesis of the compounds* ***5a-5e***

A solution of compound **4** (1.5 mmol) and proper amine (1.5 mmol) in 5 mL ACN, in presence of catalytic amount of potassium iodide and potassium carbonate was reflux overnight. The solvent was evaporated and the residue was purified with column chromatography to give final products **5a-5e**.

*N-((1-benzyl-2-(methylsulfonyl)-1H-imidazol-5-yl)methyl)benzamine* ***(5a)***

Yield: 65%, mp: 114-115°C; IR: (KBr) ν (cm^-1^): 3317 (N-H), 1127, 1308 (SO_2_). ^1^HNMR (DMSO,400 MHz) δppm: 3.32 (s, 3H, SO_2_CH_3_), 4.11 (d, *J* = 5.6 Hz, 2H, CH_2_-NH), 5.66 (s, 2H, benzyl), 6.10 (t, *J* = 5.6 Hz, 1H, NH), 6.50 (d, *J* = 7.2 Hz, 2H, H_2_, H_6_-phenyl), 6.56 (t, *J* = 7.2 Hz, 1H, H_4_-phenyl), 7.04 (t, *J* = 7.2 Hz, 2H, H_3_, H_5_-phenyl), 7.11 (m, 3H, imidazole, H_2_, H_6_-benzyl), 7.22 (t, *J* = 7.2 Hz, 1H, H_4_-benzyl), 7.40 (t, *J* = 7.2 Hz, 2H, H_3_, H_5_-benzyl). LC-MS [M+1]^+^: m/z 342, [M+23]^+^: m/z 364. Anal. Calcd for C_18_H_19_N_3_O_2_S: C, 63.32; H, 5.61; N, 12.31. Found: C, 64.00; H, 5.56; N, 12.25.

*N-((1-benzyl-2-(methylsulfonyl)-1H-imidazol-5-yl)methyl)-4-methoxybenzamine* ***(5b)***

Yield: 10%, mp: 152 °C; IR: (KBr) ν (cm^-1^): 3250 (N-H), 1150, 1360 (SO_2_), ^1^HNMR (DMSO, 400 MHz) δppm: 3.27 (s, 3H, SO_2_CH_3_), 3.76 (s, 3H, OCH_3_), 4.10 (s, 2H, CH_2_-NH), 5.70 (s, 2H, benzyl), 6.50 (d, *J* = 8.8 Hz, 2H, H_2_, H_6_-phenyl), 6.78 (d, *J* = 8.8 Hz, 2H, H_3_, H_5_-phenyl), 7.12 (dd, *J* = 8.0 Hz, 1.6 Hz, 2H, H_2_, H_6_-benzyl), 7.14 (s, 1H, imidazole), 7.36 (m, 3H, H_3_, H_4_, H_5_-benzyl). LC-MS [M+1]^+^: m/z 372, [M+23]^+^: m/z 410. Anal. Calcd for C_19_H_21_N_3_O_3_S: C, 61.44; H, 5.70; N, 11.31. Found: C, 61.52; H, 5.72; N, 11.23.

*N-((1-benzyl-2-(methylsulfonyl)-1H-imidazol-5-yl)methyl)-4-bromobenzamine* ***(5c)***

Yield: 16%, mp: 155 °C; IR: (KBr) ν (cm^-1^): 3288 (N-H), 1136, 1329 (SO_2_). ^1^HNMR (DMSO,400 MHz) δppm: 3.32 (s, 3H, SO_2_CH_3_), 4.10 (d, *J* = 6 Hz, 2H, CH_2_-NH), 5.64 (s, 2H, benzyl), 6.37 (t, *J* = 6 Hz, 1H, NH), 6.43 (d, *J* = 8.8 Hz, 2H, H_2_, H_6_-phenyl), 7.10 (m, 3H, imidazole, H_2_, H_6_-benzyl), 7.17 (d, *J* = 8.8 Hz, 2H, H_3_, H_5_-phenyl), 7.32 (t, *J* = 7.2 Hz, 1H, H_4_-benzyl), 7.38 (t, *J* = 7.2 Hz, 2H, H_3_, H_5_-benzyl). LC-MS [M+1]^+^: m/z 420, [M+3]^+^: m/z 423, [M+23]^+^: m/z 442, [M+25]^+^: m/z 444. Anal. Calcd for C_18_H_18_BrN_3_O_2_S: C, 51.43; H, 4.32; N, 10.00. Found: C, 51.48; H, 4.30; N, 9.76.

*N-((1-benzyl-2-(methylsulfonyl)-1H-imidazol-5-yl)methyl)-4-nitrobenzamine* ***(5d)***

Yield: 10%, mp: 177 °C; IR: (KBr) ν (cm^-1^): 3310 (N-H), 1110-1308 (SO_2_), 1324-1528 (NO_2_).^ 1^HNMR (DMSO,400 MHz) δppm: 3.32 (s, 3H, SO_2_CH_3_), 4.30 (d, *J* = 6 Hz, 2H, CH_2_-NH,), 5.65 (s, 2H, benzyl), 6.56 (d, *J* = 8.8 Hz, 2H, H_2_, H_6_-phenyl), 7.07 (d, *J* = 7.2 Hz, 2H, H_2_, H_6_-benzyl), 7.17 (s, 1H, imidazole), 7.31 (t, *J* = 7.2 Hz, 1H, H_4_-benzyl), 7.36 (t, *J* = 7.2 Hz, 2H, H_3_, H_5_-benzyl), 7.66 (t, *J* = 6 Hz, 1H, NH), 7.96 (d* J* =8.8 Hz, 2H, H_3_, H_5_-phenyl). LC-MS [M+23]^+^: m/z 409. Anal. Calcd for C_18_H_18_N_4_O_4_S: C, 55.95; H, 4.70; N, 14.50. Found: C, 55.91; H, 4.74; N, 14.43.

*N-((1-benzyl-2-(methylsulfonyl)-1H-imidazol-5-yl)methyl)-4-chlorobenzamine* ***(5e)***

Yield: 25%, mp: 142 °C; IR: (KBr) ν (cm^-1^): 3298 (N-H), 1140, 1333 (SO_2_). ^1^HNMR (DMSO,400 MHz) δppm: 3.32 (s, 3H, SO_2_CH_3_), 4.10 (d, *J* = 6 Hz, 2H, CH_2_-NH), 5.64 (s, 2H, benzyl), 6.32 (t,* J* = 6 Hz, 1H, NH), 6.47 (d, *J* = 8.8 Hz, 2H, H_2_, H_6_-phenyl), 7.06 (d, *J* = 8.8 Hz, 2H, H_3_, H_5_-phenyl), 7.10 (m, 3H, imidazole, H_2_, H_6_-phenyl), 7.32 (t, *J* = 7.2 Hz, 1H, H_4_-benzyl), 7.38 (t, *J* = 7.2 Hz, 2H, H_3_,H_5_-benzyl). LC-MS [M+23]^+^: m/z 398, [M+25]^+^: m/z 400. Anal. Calcd for C_18_H_18_ClN_3_O_2_S: C, 57.52; H, 4.83; N, 11.18. Found: C, 57.80; H, 4.79; N, 11.11.

*In-vitro biological activity*

The inhibitory activity of the synthesized compounds was evaluated against COX-1 and COX-2 enzymes with Cayman colorimetric-based human cyclooxygenase assay kit (item number 701050). The enzyme was incubated with inhibitors for 2 min in 0.1 M Bis-Tris/HCl buffer (pH 8.0) at 25 °C. Arachidonic acid and Celecoxib were used as substrate and reference drug respectively. All test samples were dissolved in DMSO and absorbance was read at 590 nm. The IC_50 _amounts of the novel compounds were analyzed using nonlinear regression with Dos-response inhibition parameter by the activity base software package (Program Prism, Graph Pad, SanDiego, CA).

## Results and Discussion

*Molecular Modeling Studies*

**Figure 2:**
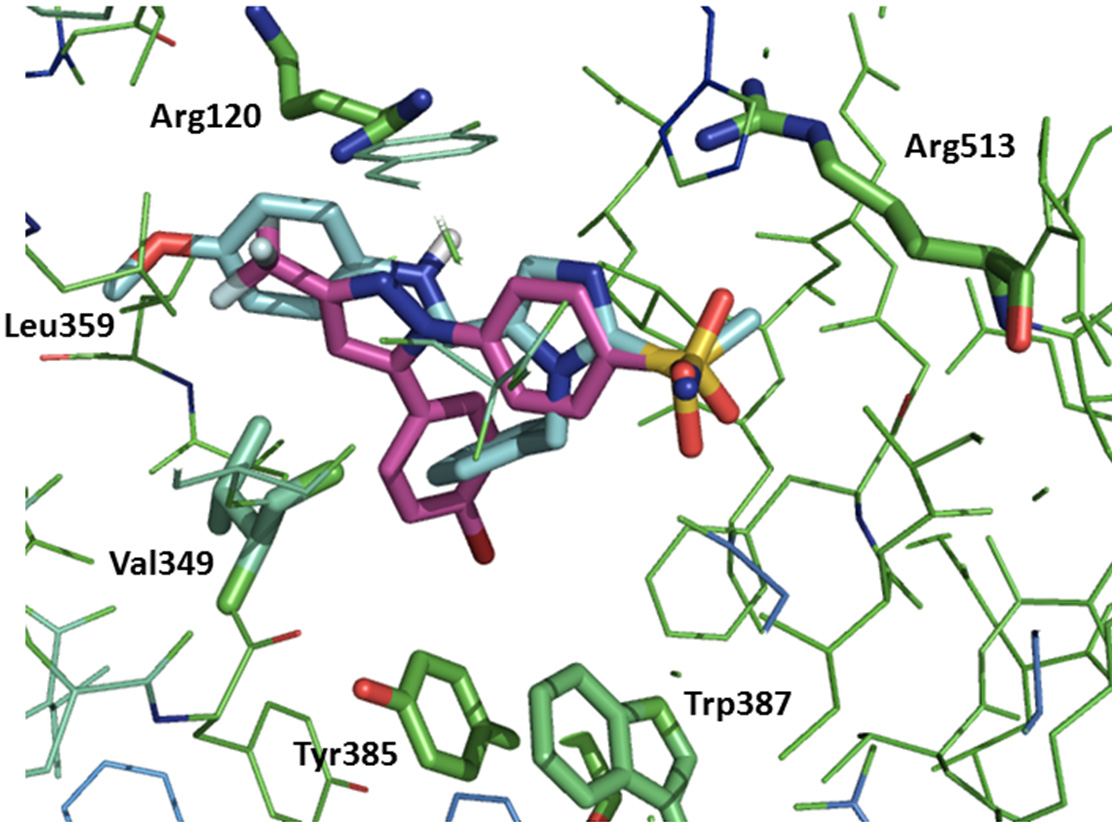
Overlay of 5b (blue) and Celecoxib (magenta) in the catalytic pocket of COX-2

To predict interaction of compounds (5a–e with COX-2 binding site docking stimulation was performed. The orientation of the most potent inhibitor **5b**, N-((1-benzyl-2-(methylsulfonyl)-1H-imidazol-5-yl)methyl)-4-methoxyaniline, along with Celecoxib in the active site of the COX-2 enzyme examined by a flexible docking experiment using AutoDock 4.0 software was observed in Figure 2. The pharmacophoric methylsulfonyl and sulfonamide groups of the **5b** and Celecoxib were in the same direction for hydrogen binding with Arg513 and also the phenyl ring of the **5b** may bind in a lipophilic pocket formed by Trp387, Tyr385, and Val349 of the active site. Additional hydrogen binding could form between NH of anilino moiety of the **5b** and Arg120. Isobutyl moiety of Leu359 may form hydrophobic interaction with methyl group of methoxy substituent of the **5b**.

*Chemistry*

**Scheme 1:**
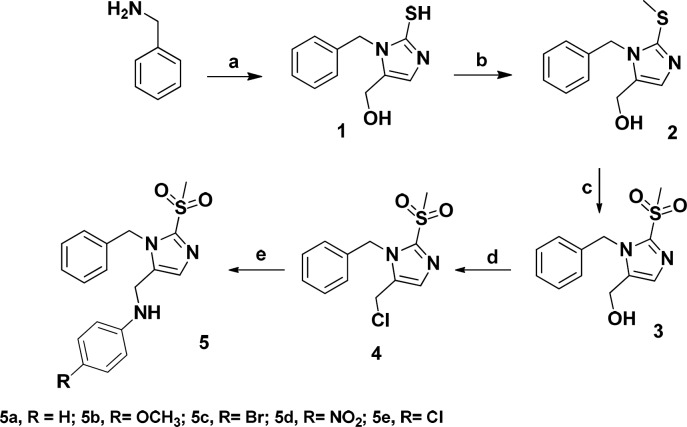
Synthesis of compounds **5a-e** Reagents and conditions: Reagents and conditions: (a) Potassium thiocyanate , dihydroxyacetone, acetic acid/ H_2_O (93/7), 55 °C, 18 h; (b) Sodium iodide, NaOH 10%, ethanol, rt, 1 h; (c) Oxone, THF/ H_2_O, rt, 24 h; (d) SOCl_2_, 70 °C, 4 h; (e) Proper amine, KI, K_2_CO_3_, ACN, 80 °C, 24 h

The designed compounds were synthesized according to Scheme 1. Benzylamine was reacted with potassium thiocyanate and dihydroxyacetone in acetic acid/water to give imidazole ring. Methyl sulfonyl moiety was afforded after S-methylation and oxidation of the thiol group. Treatment of the compound **3** with thionyl chloride followed by reaction with proper amine gave final products (**5a-5e**).

*In-vitro Biological activity*

As shown in Table 1, all of the designed compounds have acceptable COX-2 inhibitory activity with IC_50_ values in the range of 0.7-3.6 µM, while IC_50_ values of COX-1 inhibition were 78-138µM. The rank order for the contribution of substituents to the COX-2 inhibitory activity of the synthesized compounds is: OCH_3 _> Br > NO_2 _> H > Cl. Results reveal that the compound bearing methoxy (**5b**), shows the best inhibitory activity and selectivity against COX-2 with IC_50_ of 0.71 µM and selectivity index of 115 due to additional hydrophobic interaction of methoxy with Leu359. The electron withdrawing substitutes and Hydrogen at the para position of anilino ring, despite selectivity to COX-2, show no considerable priority with each other.

**Table 1:** Inhibitory activity of the imidazole derivatives against COX-1 and COX-2 enzymes

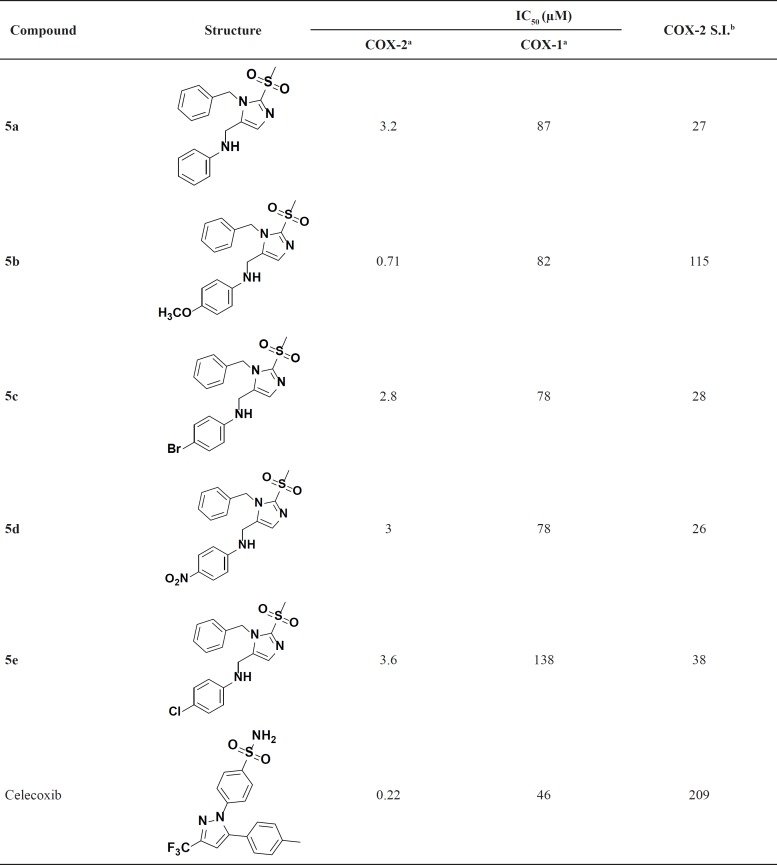

^a^The concentration of test compound produce 50% inhibition of COX-2, COX-1 enzyme, the result is the mean of two value obtained by assay of enzyme kits obtained from (Cayman colorimetric-based human cyclooxygenase assay kit Chemicals kit with item number 701050).

^b^The *in-vitro* COX-2 selectivity index (COX-1/COX-2).

## Conclusion

New imidazole-based compounds as non-classical selective cyclooxygenase-2 inhibitors were investigated by attaching the suitable pharmacophore directly to the central cyclic ring. The docking study shows the compounds fitted in the COX-2 catalytic pocket and interacted well with the active site residues. The synthesized compounds had comparable inhibitory activity to Celecoxib. Compound **5b** was found to be the most potent inhibitor with IC_50_ of 0.71 µM and selectivity index of 115 in targeting COX-2 enzyme. Finally, these structures seem to be valuable leading scaffold to design and develop novel selective COX-2 inhibitors.
